# Underground Pipeline Leakage Risk Assessment in an Urban City

**DOI:** 10.3390/ijerph17113929

**Published:** 2020-06-02

**Authors:** Shing-Yu Chen, Ming-Sheng Lin, Gary Li-Kai Hsiao, Tzu-Chi Wang, Chen-Shan Kao

**Affiliations:** 1 Program in Materials and Chemical Engineering, National United University, Miaoli 36003, Taiwan; star690720@gmail.com; 2 Environmental Incidents Specialist Team, Taipei 24765, Taiwan; mingshang0828@hotmail.com; 3 Department of Disaster Management, Taiwan Police College, Taipei 11696, Taiwan; 4 Department of Chemical Engineering and Materials Engineering, Chinese Culture University, Taipei 11114, Taiwan; tzuchiwang1@gmail.com; 5 Department of Safety, Health and Environmental Engineering, National United University, Miaoli 36003, Taiwan; jcsk@nuu.edu.tw

**Keywords:** underground pipeline, risk assessment, ALOHA, SuperGIS, emergency response

## Abstract

Underground pipeline safety is a concern among civilians in populated urban cities. Due to the potential for considerable damage from underground pipeline leakages, it is critical to identify potential risk areas. This study developed a simplified risk value using risk assessment software (ALOHA) and geography information systems (SuperGIS and Surfer) to produce potential risk maps for underground pipeline leakage in a major urban city. A risk assessment of areas affected by underground pipeline leakage was performed for vapor diffusion, thermal radiation from combustion, and overpressure from an explosion. The results are applicable to disaster management departments and agencies in highly populated cities.

## 1. Introduction

In both urban and rural areas, underground pipeline networks are hazardous infrastructure that present a high risk for fire damage and damage to ecosystems. There are many unexpected causes of leakage in underground pipelines carrying hazardous materials, and such leakages can have detrimental effects in densely populated cities, such as during a structural collapse resulting from an explosion [1]. Underground gas pipeline leakages have also been shown to negatively affect plant health and cause severe environmental damage [2,3].

Petrochemicals in particular are among the most flammable and thus most regulated chemicals, and damage to such pipelines can cause considerable destruction in fire accidents. Fires and explosions related to petrochemical pipelines have been responsible for significant injury and loss of life [4]. An investigation of nine fire accidents that occurred between 2004 and 2007 reported that pipeline damage was a major cause of explosions and ruptures, resulting in significant burn injuries and over 600 deaths at the disaster sites [5].

More obvious causes of leakage include boiling liquid expanding vapor explosion phenomena, which are a major cause of personnel and environmental hazards in gas factory accidents [6]. However, even seemingly mundane conditions, such as heavy rain, can result in petrochemical pipeline explosions due to underground pipeline leakages [7]. Deliberate damage and a lack of monitoring also pose a risk [8].

Following an underground pipeline explosion in Kaohsiung in 2014, relevant agencies focused on improving the safety of underground pipelines that transport toxic chemicals under large, densely populated urban areas [9]. Other research indicated that such measures can maintain or repair the reputations of petrochemical pipeline operators, given the importance that consumers place on accident severity when evaluating their safety and reputation [10]. In cases where buildings are affected, dark environments already make wayfinding difficult for firefighters and civilians, and the presence of chemicals hampers rescue efforts even further [11]. It is thus critical to be able to identify potential disaster areas so that appropriate precautions can be taken.

For such assessments, the present study developed a simplified risk value (SRV), which was derived using a risk assessment simulation tool named Areal Locations of Hazardous Atmospheres (ALOHA), version 5.4.7 and geography information systems including SuperGIS, version 10.1, and Surfer, version 10 to produce a potential risk map (PRM) for underground pipeline leakage in Taipei, Taiwan [12,13,14]. The SRV can be used to identify areas at risk of damage from pipeline leakages. Specifically, it shows the potential effects of vapor diffusion, thermal radiation from combustion, and overpressure from an explosion.

## 2. Method

The present study investigated underground pipelines carrying methyl tert-butyl ether (MTBE) to identify potential disaster areas in Taipei, Taiwan. MTBE is a Type 4 toxic chemical substance regulated by Taiwan’s Environmental Protection Agency. It is mainly used as an octane-free improver for unleaded gasoline. It is also an important raw material for preparing polymer-grade isobutylene. It is a colorless clarified liquid and is both flammable and toxic. Contact can cause serious damage and irritation to the skin and eyes, and it can also cause cancer.

Potential disaster areas are areas where disasters have occurred in the past and where the risk of a disaster occurring in the future is high. Producing a PRM of such areas can aid in identifying high-risk locations and provide a reference for disaster response and evacuation guidelines.

There are many risk assessment software applications for pipeline leakage simulations. The present study adopted the gas dispersion model of ALOHA because it has been widely used for risk assessment [15]. ALOHA was employed in this study to simulate areas that would be affected by an MTBE underground pipeline leakage and to calculate the SRV. Potential risk areas were evaluated for the effects of vapor diffusion, thermal radiation from combustion, and overpressure from an explosion. SuperGIS and Surfer were then employed to generate a PRM.

The pipeline leakage estimation method was combined with a risk-based inspection methodology to calculate the MTBE leakage from an underground pipeline [16].

Of the 57,160 m of pipeline in the studied underground network, only 12,656 m of pipeline was considered in this study. The size of the rupture was estimated to be 2000 mm^2^, based on data from the 2014 Kaohsiung underground pipeline explosion. The leakage of MTBE from the underground pipeline was estimated to be approximately 115,113 to 124,145 kg of mass.

The level of concern (LOC) estimation was based on the Emergency Response Planning Guidelines (ERPG) of the American Industrial Hygiene Association [17]. General risk is defined as a result of the consequences of a hazard, which is usually defined as the number of deaths. A complete risk assessment would be impractical because of the wide distribution of the underground pipeline facilities and variation in the pressure and diameter of pipelines across the network. Therefore, this study defined the SRV as follows:

SRV = hazard radius × frequency(1)

where the hazard radius was measured in kilometers and the frequency was defined as the number of occurrences per year.

ERPG-3 refers to the maximum allowable concentration of a toxic airborne chemical that a person can be exposed to for approximately 1 h without threatening their life. This study used an ERPG-3 thermal radiation value of 12.5 kW/m^2^ and overpressure of 0.5 psi as the LOC in order to evaluate the toxic effects of vapor dispersion leaking into the environment, thermal radiation from combustion, and overpressure generated by an explosion. The pressure of the MTBE underground pipeline was estimated to be 23.5 kg/cm^2^ at the start location and 20.4 kg/cm^2^ at the end location. The leak duration was set to 30 min. Table 1 shows the simulation parameters.

For vapor dispersion, thermal radiation from combustion, and overpressure from an explosion, the hazard radius correlated positively with the hazard consequences in the ALOHA simulation. According to the American Institute of Chemical Engineers, the frequency of pipeline failure by rupture under normal operation is 0.000235 times per year for metal pipelines. Therefore, the SRV identifies areas affected by possible damage from vapor dispersion, thermal radiation from combustion, and overpressure from an explosion every year. Combined with GIS data, the relative potential risks of affected areas were obtained from the SRV on a PRM.

## 3. Results and Conclusions

**Figure 1:**
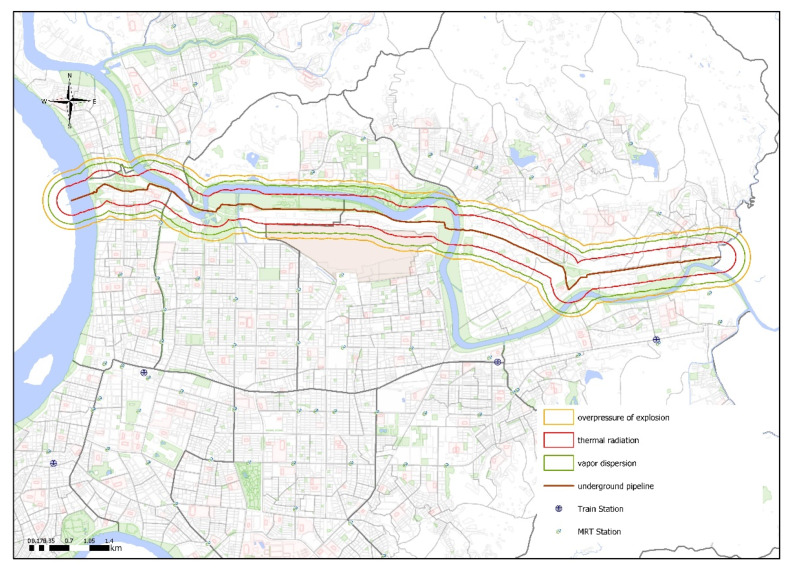
Hazard radius for vapor diffusion, thermal radiation, and overpressure due to pipeline leakage.

The hazard radius from underground pipeline leakage to nearby affected areas was determined to be 431 m for vapor dispersion, 271 m for thermal radiation from combustion, and 527 m for overpressure from an explosion. Figure 1 shows the hazard radius overlaid on the PRM generated using SuperGIS and Surfer.

**Figure 2:**
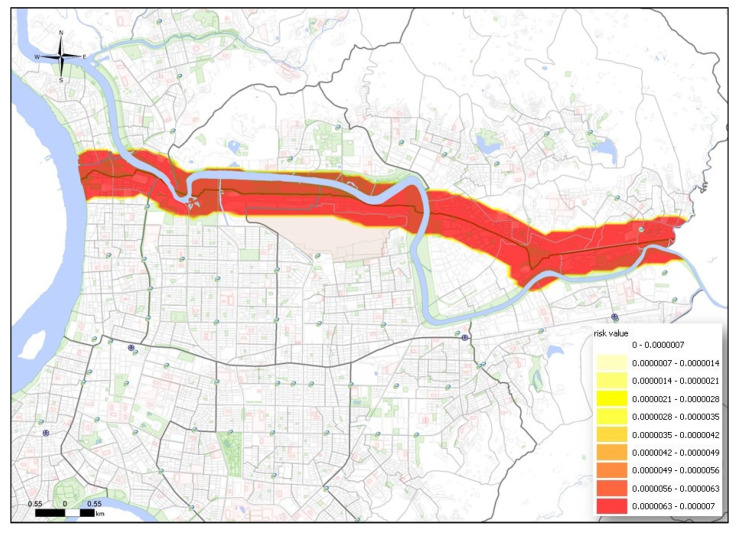
PRM of vapor diffusion to affected areas by underground pipeline leakage.

**Figure 3:**
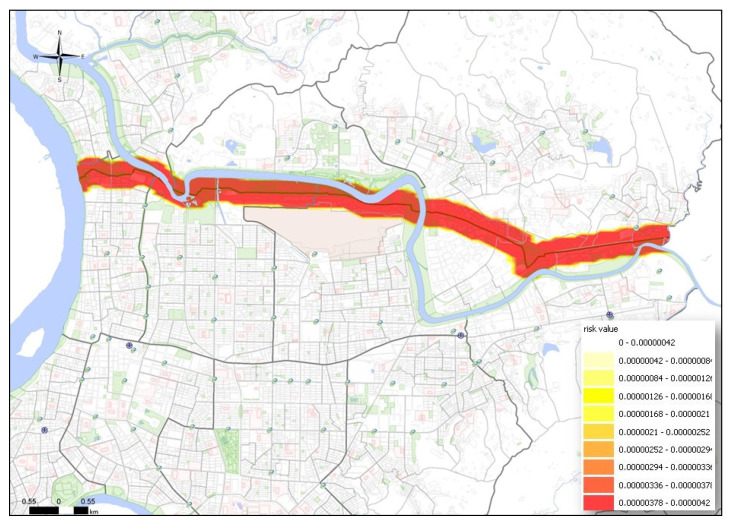
PRM of thermal radiation to affected areas by underground pipeline leakage.

**Figure 4:**
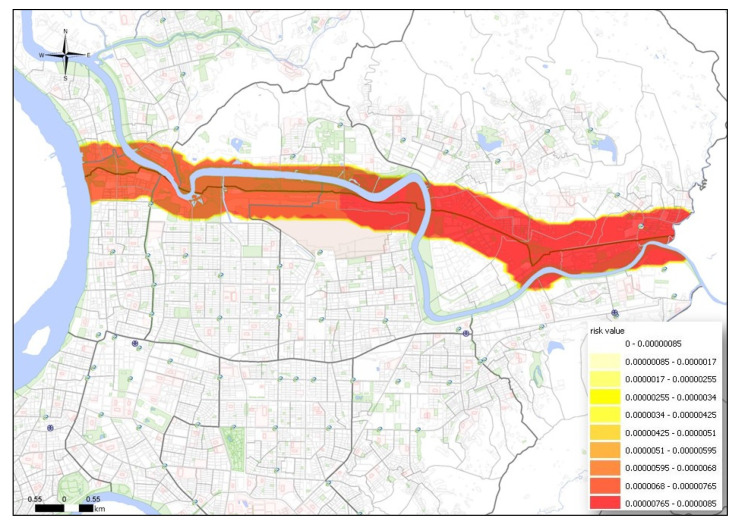
PRM of overpressure from an explosion to affected areas by underground pipeline leakage.

The MTBE underground pipeline spans the entire urban area, with a total length of 12,656 m. The underground pipeline runs through 5 districts and 12 villages. Following the ALOHA simulation, the hazard radius for vapor diffusion, thermal radiation from combustion, and overpressure from an explosion were converted into an SRV. As shown in Figure 2, Figure 3 and Figure 4, vapor dispersion affected 6 districts and 27 villages, thermal radiation from combustion affected 6 districts and 17 villages, and overpressure from an explosion affected 6 districts and 30 villages.

The ALOHA simulation of an MTBE underground pipeline leakage was conducted to explore the potential risk areas for vapor diffusion, thermal radiation from combustion, and overpressure from an explosion. An SRV was derived from the combination of the hazard radius and frequency of consequence, and then mapped via GIS tools to produce a PRM. The results are applicable to disaster management departments and agencies. Discussions should be held to determine how these findings might affect urban planning, particularly regarding the location and population distribution within the hazard area. Vulnerable locations include schools, governmental agencies, medical facilities, mass rapid transit stations, and oil or gas stations should be evaluated prior to others. Areas at risk of being affected by an MTBE underground pipeline leakage should be examined to determine the appropriate evacuation planning, including the selection of an appropriate evacuation site, evacuation routes, intersection control, and assembly locations. All these considerations need to be well planned in advance to ensure a more timely emergency response.

## Figures and Tables

**Table 1:** ALOHA simulation parameter settings.

Condition	Setting
Duration of leakage	30 min
Wind speed	1.0 m/s
Ambient temperature	28 °C
Humidity	75%
Size of rupture	2000 mm^2^
Leak form	Continuous leakage
LOC of toxicity	5000 ppm
LOC of thermal radiation	12.5 kW/m^2^
LOC of overpressure by explosion	0.5 psi
